# A Call for Help: A Retrospective National Study of Self-Inflicted Trauma Among American Indians and Alaskan Natives

**DOI:** 10.7759/cureus.53624

**Published:** 2024-02-05

**Authors:** Conor S Roche, Hilla I Sang, Mentor Ahmeti

**Affiliations:** 1 General Surgery, University of North Dakota School of Medicine and Health Sciences, Grand Forks, USA; 2 General Surgery, Sanford Health, Fargo, USA; 3 Statistics, Sanford Health, Fargo, USA; 4 Trauma and Acute Care Surgery, Sanford Health, Fargo, USA

**Keywords:** public health, ntdb, self-inflicted, trauma, american indian

## Abstract

Background

Self-inflicted injury accounts for approximately 312,000 emergency department visits annually. American Indians/Alaskan Natives (AIAN) have significantly higher rates of suicide. The National Trauma Data Bank (NTDB) was analyzed for the incidence of self-inflicted trauma.

Methods

Data were obtained from the NTDB 2012-2017. Patients were selected using ICD codes for self-inflicted trauma. Categorical and continuous variables were tested for significance.

Results

AIAN patients accounted for 1,176 of the 78,668 patients. The AIAN patients were younger, had lower injury severity score (ISS) scores, were more female, utilized Medicaid more frequently, were more likely to present with a cut or piercing injury, and had higher rates of positive alcohol and drug tests. AIAN patients had shorter lengths of stay in the ICU and overall hospital stay.

Conclusion

Despite a higher rate of suicide completion, the AIAN population had lower rates of presentation to the hospital and lower ISS scores. AIAN patients were younger, had higher rates of drug use, and utilized cutting/stabbing. This discrepancy could indicate a physical manifestation of a “call for help”.

## Introduction

According to the Centers for Disease Control, self-inflicted injuries account for over a quarter million emergency department visits annually [1]. A self-inflicted injury resulting from an attempted suicide in adolescents increases the risk of completion suicide by 20 to 50 times [2,3]. Suicide has become the overall tenth most common cause of death while it is the second most common cause of death in individuals 10 to 34 years old, the fourth most common cause of death in those from 34-54 years old, and the fifth most common cause of death in people 45 to 54 years old [4]. Even with those statistics, the American Indian/Alaskan Native (AIAN) population has a significantly higher rate of suicide compared to their non-American Indian counterparts. American Indians account for around 2.5% of the US population but average 28 per 100,000 people for suicide completion rate as compared to 10 per 100,000 in the non-American Indian population [5]. The reasons for this are likely multifactorial but suicide has been associated with PTSD and historical trauma while AIAN youth may be more likely to have suicide attempts when their social network involves people who use alcohol or drugs [6,7]. Using nationally collected data from the National Trauma Data Bank, we sought to better look at how hospitals are encountering individuals with self-inflicted trauma. Due to the increased rates of suicide found in the American Indian population, we sought to study the differences between American Indian population admissions compared to non-American Indian population. We hypothesized that the American Indian population who initially survived a self-inflicted trauma would present to the hospitals more frequently and with more severe injuries than their non-American Indian counterparts. The purpose of this research is to study the epidemiologic data, prevalence, and characteristics of self-inflicted harm in the American Indian population from the standpoint of national hospital data.

## Materials and methods

This study is a retrospective review of the NTDB for the years 2012-2017 and includes all patients whose intent of injury was marked as “self-inflicted”. The database is a national data bank of voluntarily submitted data from individual hospitals. The data bank inclusion and exclusion criteria are based on the International Statistical Classification of Diseases, Clinical Modification, diagnoses codes, and admission data. The NTDB is an incident-based record, which means that there may be duplication of a specific patient included in the databank [8]. The deidentified data collected by the NTDB was obtained and the patients to be included in the study were identified. Inclusion criteria were patients in the NTDB who were identified as having self-inflicted by the respective ICD9 codes (E950-E959). Exclusion criteria were patients who had missing race and who did not meet the ICD9 codes listed for the inclusion criteria. Demographic characteristics included age, sex, race, and insurance status. Race was categorized into a binary variable identifying patients as American Indian/Alaskan Native (AIAN) and those who were not identified as AIAN. Insurance status was categorized into self-pay, Medicaid, Medicare, private/commercial insurance, Blue Cross/Blue Shield, Workers Compensation, other government, and other/unknown. Injury severity score (ISS) was categorized as 9 and under, 10-15, 16-24, and 25 and over.

Variables of interest (injury type and mechanism, substance use, length of stay, and discharge disposition) were summarized using mean and standard deviation (SD) for continuous variables and counts and percentages for categorical variables. The chi-squared test of independence was conducted on the categorical variables while the Kruskal Wallis H-test was conducted on continuous variables. After the removal of duplicate cases, a total of 78,669 patients were included in the study. Data were compiled, organized, and analyzed using RStudio software (RStudio Team (2020)), and the statistical significance level was set at 0.05.

## Results

The total number of patients included in the study using our inclusion and exclusion criteria was 78,668 patients. AIAN patients accounted for 1.5% of our study population (1,176 patients). Throughout the data, several areas of analysis were identified as statistically significant differences between AIAN and non-AIAN patients. Demographic differences as shown in Table 1 indicated that AIAN patients presenting with self-inflicted trauma are younger than non-AIAN patients (30.9 vs. 38.7 years, respectively, p < .001), female (28.7% vs. 25.9%, respectively, p = .032), and have Medicaid as their primary payer (40.3% vs. 21.4%, respectively, p<.001). Non-AIAN patients are more likely to be covered under private/commercial insurance (23.9%) or self-pay (18.4%).

**Table 1:** Demographic, payment, Injury severity score (ISS), substance use, length of stay, disposition 1. Kruskal-Wallis rank sum test 2. Pearson’s chi-squared test AIAN: American Indians/Alaskan Natives; ISS: injury severity score; LOS: length of stay

	Non-AIAN (N=77492)	AIAN (N=1176)	Total (N=78668)	p value
Age				< 0.01^1^
Mean (SD)	38.70 (16.92)	30.92 (12.07)	38.58 (16.88)	
Median (Q1, Q3)	36.00 (25.00, 51.00)	27.50 (22.00, 37.25)	36.00 (25.00, 51.00)	
Min - Max	1.00 - 89.00	4.00 - 84.00	1.00 - 89.00	
Gender				0.032
Female	20056 (25.9%)	337 (28.7%)	20393 (25.9%)	
Male	57416 (74.1%)	839 (71.3%)	58255 (74.1%)	
Mode of Hospital Bill Payment				< 0.01^2^
Self-Pay	14260 (18.4%)	138 (11.7%)	14398 (18.3%)	
Private/Commercial Insurance	18556 (23.9%)	136 (11.6%)	18692 (23.8%)	
Medicare	10646 (13.7%)	48 (4.1%)	10694 (13.6%)	
Medicaid	16585 (21.4%)	474 (40.3%)	17059 (21.7%)	
Blue Cross/Blue Shield	2496 (3.2%)	17 (1.4%)	2513 (3.2%)	
Workers Compensation	73 (0.1%)	0 (0.0%)	73 (0.1%)	
Other Government	4371 (5.6%)	262 (22.3%)	4633 (5.9%)	
Other/Unknown	10505 (13.6%)	101 (8.6%)	10606 (13.5%)	
ISS level				< 0.01^2^
9 and under	47995 (62.0%)	900 (76.7%)	48895 (62.2%)	
10 to 15	7379 (9.5%)	108 (9.2%)	7487 (9.5%)	
16 to 24	7444 (9.6%)	81 (6.9%)	7525 (9.6%)	
25 and over	14611 (18.9%)	85 (7.2%)	14696 (18.7%)	
Alcohol Use				< 0.01^2^
No (confirmed by test)	25828 (35.2%)	232 (20.5%)	26060 (35.0%)	
No (not tested)	18861 (25.7%)	240 (21.2%)	19101 (25.6%)	
Other/Unknown	3786 (5.2%)	44 (3.9%)	3830 (5.1%)	
Yes (confirmed by test [beyond legal limit])	13737 (18.7%)	462 (40.8%)	14199 (19.1%)	
Yes (confirmed by test [trace levels])	11138 (15.2%)	154 (13.6%)	11292 (15.2%)	
Drug Use				< 0.01^2^
No (confirmed by test)	12713 (20.0%)	190 (19.7%)	12903 (20.0%)	
No (not tested)	26667 (41.9%)	377 (39.1%)	27044 (41.8%)	
Other/Unknown	6584 (10.3%)	43 (4.5%)	6627 (10.2%)	
Yes (confirmed by test)	17743 (27.9%)	354 (36.7%)	18097 (28.0%)	
Hospital LOS				< 0.01^3^
Mean (SD)	5.95 (11.14)	4.33 (6.98)	5.93 (11.09)	
ICU LOS				0.013
Mean (SD)	5.33 (8.08)	4.48 (6.44)	5.32 (8.06)	
ED Discharge Disposition				< 0.01^2^
Deceased/expired	5710 (7.4%)	26 (2.2%)	5736 (7.3%)	
Floor bed (general admission, non specialty unit bed)	16151 (20.9%)	294 (25.0%)	16445 (21.0%)	
Home	3001 (3.9%)	43 (3.7%)	3044 (3.9%)	
Intensive Care Unit (ICU)	21036 (27.2%)	251 (21.4%)	21287 (27.1%)	
Left against medical advice	81 (0.1%)	3 (0.3%)	84 (0.1%)	
Observation unit	1341 (1.7%)	47 (4.0%)	1388 (1.8%)	
Operating Room	19601 (25.4%)	342 (29.1%)	19943 (25.4%)	
Other (jail, institutional care facility, mental health, etc)	2589 (3.4%)	40 (3.4%)	2629 (3.4%)	
Other/Unknown	1633 (2.1%)	30 (2.6%)	1663 (2.1%)	
Telemetry/step-down unit (less acuity than ICU)	2922 (3.8%)	34 (2.9%)	2956 (3.8%)	
Transferred to another hospital	3179 (4.1%)	64 (5.5%)	3243 (4.1%)	
					Hospital Discharge Disposition				< 0.01^2^
Another type of institution not defined elsewhere	5060 (6.8%)	86 (7.5%)	5146 (6.8%)	
Another type of rehabilitation or long term	5084 (6.8%)	76 (6.6%)	5160 (6.8%)	
Court/law enforcement	1085 (1.5%)	18 (1.6%)	1103 (1.5%)	
Deceased/Expired	8744 (11.7%)	53 (4.6%)	8797 (11.6%)	
Home or self-care (routine discharge)	22091 (29.6%)	496 (43.2%)	22587 (29.8%)	
Home under care of organized home health service	1396 (1.9%)	7 (0.6%)	1403 (1.9%)	
Hospice care	383 (0.5%)	0 (0.0%)	383 (0.5%)	
Inpatient rehab or designated unit	1716 (2.3%)	13 (1.1%)	1729 (2.3%)	
Intermediate Care Facility (ICF)	765 (1.0%)	7 (0.6%)	772 (1.0%)	
Left against medical advice or discontinued care	553 (0.7%)	9 (0.8%)	562 (0.7%)	
Long Term Care Hospital (LTCH)	672 (0.9%)	9 (0.8%)	681 (0.9%)	
Other/Unknown	11900 (16.0%)	151 (13.2%)	12051 (15.9%)	
Psychiatric hospital or psychiatric distinct part unit of a hospital	9650 (12.9%)	170 (14.8%)	9820 (13.0%)	
Short-term general hospital for inpatient care	3722 (5.0%)	28 (2.4%)	3750 (5.0%)	
Skilled Nursing Facility (SNF)	1728 (2.3%)	24 (2.1%)	1752 (2.3%)	

Chi-square testing showed that the mechanism of injury used between AIAN and non-AIAN patients was statistically different (p < .001), as shown in Table 2. Specifically, AIAN patients were more likely to present with a cut or piercing injury (48.2% vs. 33.4% among non-AIAN) while non-AIAN patients were more likely to present with a firearm injury (24.3% vs. 14.2% among AIAN). Substance abuse was more prevalent among AIAN patients. AIAN patients were more likely than non-AIAN to have consumed alcohol beyond the legal limit (40.8% vs. 18.7%, p <.001) and to have tested positive for drug use (36.7% vs. 27.9% for non-AIAN patients, p<.001). ISS was also statistically significant with non-AIAN patients more often presenting with ISS >25 (18.9% vs. 7.2%) and AIAN patients more often presenting with ISS <9 (76.7% vs. 62%).

**Table 2:** Mechanism of injury 1. Pearson’s chi-squared test

	Non-AIAN (N=77492)	AIAN (N=1176)	Total (N=78668)	p value
Self-Inflicted Injury Mechanism				< 0.01^1^
Burn	1093 (1.4%)	8 (0.7%)	1101 (1.4%)	
Cut/pierce	25900 (33.4%)	567 (48.2%)	26467 (33.6%)	
Drowning/submersion	50 (0.1%)	0 (0.0%)	50 (0.1%)	
Fall	6732 (8.7%)	57 (4.8%)	6789 (8.6%)	
Firearm	18862 (24.3%)	167 (14.2%)	19029 (24.2%)	
Motor vehicle/Transportation	1818 (2.3%)	16 (1.4%)	1834 (2.3%)	
Poisoning	752 (1.0%)	8 (0.7%)	760 (1.0%)	
Struck by, against an object	181 (0.2%)	4 (0.3%)	185 (0.2%)	
Suffocation	2890 (3.7%)	56 (4.8%)	2946 (3.7%)	
Other/Unknown	19214 (24.8%)	293 (24.9%)	19507 (24.8%)	

Statistically significant differences were also revealed for mean length of stay and discharge disposition. AIAN patients had shorter length of stay than non-AIAN in the ICU (4.48 vs. 5.33 days, p = .01) and overall hospital stay (4.33 vs. 5.95 days, p<.001). Non-AIAN patients with a self-inflicted injury were more likely to have died in the ED (7.4% vs 2.2%, p<.01) or to be admitted to an intensive care unit (27.2% vs. 21.4%, p<.01), whereas AIAN patients were more likely to be admitted to a general admission bed (25% vs. 20.9%, p<.01). In terms of overall hospital stay, non-AIAN patients had a higher incidence of expiring (11.7% vs. 4.6% of AIAN patients, p<.01), whereas AIAN were more likely to be routinely discharged to home or self-care (43.2% vs. 29.6 for non-AIAN, p<.01). Differences for ED discharge and hospital discharge were statistically significant at the p<.001 level.

## Discussion

The AIAN population only accounted for 1.5% of the study data set, yet accounted for 2.5% of the US population. Although studies showed AIAN suicide rates higher than their non-AIAN counterparts, this study showed that the AIAN population presents to national hospitals at lower rates, less severe self-inflicted injuries (based on ISS scores), and resulting in a lower rate of mortality [5]. The reasons for this could originate from a variety of causes, such as a higher completion rate in the field (not able to be fully evaluated in this study), lack of access to emergency medical services, and distance from trauma centers due to the remote location of reservations [9]. Additionally, our study showed significant differences in the AIAN versus non-AIAN population in that the AIAN patients present with less severe injuries, at younger ages, more female, increased rates of substance abuse, utilized cutting and stabbing more frequently, used federal assistance healthcare more frequently to pay for their admission, and had shorter ICU and hospital stays. With these findings, it is the opinion of the authors that AIAN patients present with self-inflicted injuries and are exhibiting a physical manifestation of a “call for help”. This is a novel epidemiological study on national hospital-reported data for self-inflicted injuries in the American Indian population. There has been some data that shows even non-suicidal self-injurious behavior poses risks for subsequent suicide attempts and deaths in the AIAN population, especially nonsuicidal self-injury (NSSI) among young [10]. Thus, self-inflicted trauma in the AIAN population that presents to US hospitals may provide a unique time for intervention and prevention of further self-injurious behavior and suicide. Some limitations of our study include unclear causation of the decrease in the rate of the American Indian population in the data set. The decreased rate is likely multifactorial due to the nature of the data bank as well as various social and economic challenges that face the AIAN population. Another is the limitation of the NTDB data set, which includes patients that have no race documented and potentially, in some cases, misidentified race. This study helps further characterize self-inflicted trauma admission to the self-inflicted harm and identify possible times for intervention. The data reported here could be used to help direct and improve public health initiatives, mental health awareness, and social work consults, as well as shine a light on healthcare access to care for the AIAN population [11]. While suicide attempts have been shown to be the highest risk factor of completion suicide, some studies have described potential protective factors in AIAN violence and injury that include “family and nonfamily connectedness, self-efficacy, cultural connectedness, and positive opportunities” [12]. Due to the discrepancy between CDC and NTDB data, findings from the data bank do not accurately reflect the overall differences in the mechanism of injury and ISS with self-inflicted injury because hospitals do not have a chance to see the severely injured self-inflicted injuries, many of those likely completion suicides of AIAN population. Healthcare initiatives should focus on improving suicide prevention services and mental healthcare for the AIAN populations, which could include new technologies like telehealth [13]. Future investigations should focus on more localized and regional data, which may provide more sensitive information, especially in areas with a high AIAN population density. Further investigations into the reasons for the lack of data in the NTDB may help explain some of the concerning findings in this study. Finally, continued research into American Indian mental health, suicide, and access to healthcare should be paramount [14,15].

## Conclusions

There are several areas where there are inequalities regarding the healthcare of AIAN patients. Our study of a large national trauma database showed that despite a higher rate of suicide completion, the AIAN population had lower rates of presentation to the hospital and lower ISS scores. AIAN patients were younger, had higher rates of drug use, and utilized cutting/stabbing more than their counterparts. This discrepancy could indicate a physical manifestation of a “call for help” and shows that further investigation into this subject is warranted.
